# Practical Notes on Diagnosis and Treatment

**Published:** 1910-12-31

**Authors:** 


TREATMENT.

### PRACTICAL NOTES ON DIAGNOSIS AND TREATMENT.

### False Angina Pectoris.

MOST cases of false angina can be distinguished by the facts that the attack supervenes spontaneously, that it lasts longer than in true angina, that it is accompanied by restless movement, that it tends to recur again and again before finally subsiding, and that there usually co-exists throbbing of the abdominal aorta, mobility of one kidney, vasomotor manifestations, and other evidences of nerve instability.—*Dr. Guthrie Rankin.*

### Washing out the Bladder.

No one should attempt to wash out a bladder if he is not fully prepared to make use of all the resources of aseptic surgery. Unless carefully carried out, fresh forms of infection may be introduced into the inflamed bladder and the original bacteria will not be killed. Of all the antiseptics which are introduced into the human bladder, I think that silver nitrate in distilled water is by far the most useful and most efficient. Begin with $\frac{1}{10}$ gr. to the ounce, and gradually increase the strength until it causes slight pain and discomfort. Do not forget to get rid of all traces of urine in the bladder by washing out with boric lotion, or the silver nitrate will be neutralised and rendered inert.—*Mr. C. B. Lockwood.*

### The Abdomen in Typhoid.

THE abdomen in typhoid is seen in two different varieties, the hard or tympanic and the soft or doughy. It is clear that when a certain degree of tympanites is present the close application of the intestinal coils to each other is more likely to be to some extent a safeguard, since extravasation tends to be limited, and perforation is somewhat less probable; tympanites must, therefore, not be regarded as altogether harmful. The outlook is distinctly less favourable when perforation is met with in the second class of abdomen.—*Dr. Sidney Phillips.*

### Atrophic Rhinitis.

THIS disease is due to a series of degenerative changes of the nasal mucous membrane, resulting in abnormal patency of the nasal fossæ and accompanied by a purulent or muco-purulent secretion, which tends to collect, dry into crusts, and undergo putrefaction, and thus to produce the most distressing symptom of the disease—namely, ozæna, by which name the disease itself is often designated. The retention, drying, and putrefaction of the discharge is chiefly due to the abnormal roominess of the nasal cavities, which renders "blowing the nose" ineffectual for the purposes of cleansing.—*Mr. C. A. Parker.*

